# Look Who’s Talking NOW! Parentese Speech, Social Context, and Language Development Across Time

**DOI:** 10.3389/fpsyg.2017.01008

**Published:** 2017-06-20

**Authors:** Nairán Ramírez-Esparza, Adrián García-Sierra, Patricia K. Kuhl

**Affiliations:** ^1^Department of Psychological Sciences, University of Connecticut, StorrsCT, United States; ^2^Department of Speech, Language, and Hearing Sciences, University of Connecticut, StorrsCT, United States; ^3^Institute for Learning and Brain Sciences, University of Washington, SeattleWA, United States

**Keywords:** LENA, parentese speech, motherese, baby talk, language development, social interactions, longitudinal

## Abstract

In previous studies, we found that the social interactions infants experience in their everyday lives at 11- and 14-months of age affect language ability at 24 months of age. These studies investigated relationships between the speech style (i.e., parentese speech vs. standard speech) and social context [i.e., one-on-one (1:1) vs. group] of language input in infancy and later speech development (i.e., at 24 months of age), controlling for socioeconomic status (SES). Results showed that the amount of exposure to parentese speech-1:1 in infancy was related to productive vocabulary at 24 months. The general goal of the present study was to investigate changes in (1) the pattern of social interactions between caregivers and their children from infancy to childhood and (2) relationships among speech style, social context, and language learning across time. Our study sample consisted of 30 participants from the previously published infant studies, evaluated at 33 months of age. Social interactions were assessed at home using digital first-person perspective recordings of the auditory environment. We found that caregivers use less parentese speech-1:1, and more standard speech-1:1, as their children get older. Furthermore, we found that the effects of parentese speech-1:1 in infancy on later language development at 24 months persist at 33 months of age. Finally, we found that exposure to standard speech-1:1 in childhood was the only social interaction that related to concurrent word production/use. Mediation analyses showed that standard speech-1:1 in childhood fully mediated the effects of parentese speech-1:1 in infancy on language development in childhood, controlling for SES. This study demonstrates that engaging in one-on-one interactions in infancy and later in life has important implications for language development.

## Introduction

Language input has substantial impact on language learning in children (e.g., Huttenlocher et al., 1991; Hart and Risley, 1995, 1999; Rowe, 2012; Ramírez-Esparza et al., 2014, 2017; for a review see Hoff, 2006), and various characteristics of that input have been associated with language learning outcomes. Speech style is one important aspect of language input early in life. When caregivers speak directly to their infants and young children, they often use a distinct speech style, which is commonly called “baby talk” (Solomon, 2011) or “parentese speech” (Ramírez-Esparza et al., 2014, 2017). Parentese is a speech style that is characterized by higher pitch, slower tempo, and exaggerated intonation contours (Fernald, 1985; Grieser and Kuhl, 1988). It contains particularly good phonetic exemplars – sounds that are clearer, longer, and more distinct from one another – acoustically “exaggerated” when compared to standard speech (Kuhl et al., 1997; Burnham et al., 2002). Parentese is also associated with exaggerated articulatory gestures and social affect (Weikum et al., 2007), and infants have been found to prefer parentese speech over standard speech (for a review see Cristia, 2013).

Previous work has demonstrated that parentese speech is beneficial to young language learners early in the process of language acquisition (Fernald, 1985; Karzon, 1985; Fernald and Kuhl, 1987; Hirsh-Pasek et al., 1987; Kemler Nelson et al., 1989). Liu et al. (2003) analyzed parentese speech directed to 6–8 and 10–12 month old infants in a laboratory setting. The results showed that the acoustic exaggeration in parentese speech is associated with the infant’s ability to discriminate difficult computer-synthesized speech contrasts, an ability that has been shown to increase with age (Liu et al., 2003). Other studies have shown that parentese speech influences early spoken word recognition. Singh et al. (2009) familiarized 7 and 8 month old infants with two unknown words, one produced in parentese speech and the other produced in standard speech. Recognition of these words in sentence context was tested 24 h later. The results showed that the infants were able to recognize words familiarized using parentese speech more effectively than words familiarized using standard speech.

Social context is another important aspect of language input that impacts early language learning (Kuhl, 2007). Previous studies in the laboratory have demonstrated that social responses to infants’ early babbling affect the quantity and quality of their vocalizations (Goldstein et al., 2003; Goldstein and Schwade, 2008). In addition, second language learning in infants is heavily dependent on social interaction, at the word and phonetic level (Kuhl et al., 2003; Kuhl, 2007; Conboy and Kuhl, 2011), and social responses during second language exposure sessions predict the degree to which individual children learn phonemes and words (Conboy et al., 2015; Kuhl, 2011). In more naturalistic settings, Ramírez-Esparza and colleagues report that there is an advantage to speech directed toward infants in a one-on-one (1:1) social context, allowing adults to respond to infants contingently in bi-directional interactions (Ramírez-Esparza et al., 2014, 2017).

### Language Learning As a Function of Speech Style and Social Context in Prelinguistic Infants

The social context of language input has been associated with language learning (e.g., Goldstein et al., 2003; Braarud and Stormark, 2008; Goldstein and Schwade, 2008; Smith and Trainor, 2008). For example, the 1:1 social context has been shown to promote the occurrence of contingent bi-directional interactions and parentese speech. When infants listen to parentese speech in a 1:1 setting, they are able to direct their attention to the sounds coming from the caregivers and, in turn, the caregivers are sensitive to the positive response from their infants. Smith and Trainor (2008) evaluated contingent bi-directional interactions in 4-month-old infants. The researchers physically separated the mother and child, placing them in different rooms. Mothers watched their infants in real time on a video display and were instructed to speak to their infants and try to make them happy. However, unbeknownst to the mothers, infants did not hear their mothers’ voices; instead the infants reacted to an experimenter (out of view of the camera) who interacted with or ignored the infant. In one condition the experimenter positively engaged the infant when the mother used parentese speech (e.g., experimenter would talk to the infant, use his/her name, and make other statements to arouse the infant), and in another condition the experimenter positively engaged the infant when the mother used standard speech. The results showed that when infants were stimulated in response to parentese speech, mothers tended to increase the pitch of parentese speech, whereas when infants were stimulated in response to standard speech, there were no changes in the pitch used by the mothers. This indicates that mothers were sensitive to the feedback produced by the infants and that this in turn affected the mothers’ pitch. Similar findings with 2- to 4-month old infants were reported by Braarud and Stormark (2008) in a study of remote mother and child interaction using closed-circuit TV systems. In one condition the infant and mother interacted in real time, and in a second condition the interactions were decoupled by presenting the responses recorded from the previous interactions to the mothers and infants. The results showed that mothers used significantly more parentese speech during the real-time interaction than during the decoupled interaction.

Other studies also suggest that contingent bi-directional interaction relates to language learning, independent of parentese speech (Goldstein et al., 2003; Goldstein and Schwade, 2008). Goldstein et al. (2003) instructed caregivers to respond to their 8-month-old infants’ babbling behavior by smiling at, moving closer to, and touching their infants. After a baseline period of this interaction, half of the caregivers were instructed to continue responding contingently to their infants’ vocalizations by smiling at, moving closer to, and touching their infants. The reactions of the other half of the caregivers (“yoked” controls) were controlled by the experimenter’s instructions and timed to match the vocalizations produced by infants assigned to the contingent group; that is, the responses of yoked control caregivers were identical to contingent group caregivers, but were not contingent on their own infants’ vocalizations. The results demonstrated that infants in the contingent group not only produced more vocalizations than infants in the yoked group, but that their vocalizations were more mature and adult-like when compared with those of the yoked group. In a follow-up study, Goldstein and Schwade (2008) had caregivers respond to their 9.5-monthold infants’ babbling in two contingent conditions by producing vocalizations containing either fully resonant vowels or words (i.e., providing exposure to consonant–vowel alternation). Mothers in yoked conditions also produced either vowels or words, but their reactions were timed to coincide with vocalizations of infants in the contingent groups. Infants in both contingent conditions increased production of the type of speech sounds they heard from their caregivers. Infants exposed to fully resonant vowels, increased their proportion of resonant vowels, and infants exposed to words increased their proportion of consonant-vowel syllables. Yoked infants, did not change the phonological characteristics of their babbling.

Recently, we reported the utilization of an innovative approach to understand the effects of both speech style and social context on language learning in monolingual families (Ramírez-Esparza et al., 2014). Specifically, families with 11 or 14-month old infants were audio recorded for approximately 32 h across 4 days as they went about their lives, using a language environment analysis system (LENA Foundation, Boulder Colorado). Recordings were coded in terms of parentese speech and standard speech in two social contexts, speech directed to the infant while she/he is alone with the speaker (i.e., 1:1 social context), or speech directed to the infant while she/he is with a group of adults (group social context). Because previous work has shown strong relationships between SES and language learning (e.g., Hart and Risley, 1995, 1999; Hoff, 2003; Rowe, 2008; Huttenlocher et al., 2010), analyses controlled for this factor. The goal was to assess the relationship between language input and language development, examining both speech style and social context, above and beyond SES. We found that increased exposure to parentese speech in a 1:1 social context in infancy was associated with more frequent concurrent vocalizations and increased productive vocabulary at 24 months (as measured by MacArthur-Bates Communicative Development Inventory or CDI; Fenson et al., 2007), controlling for SES. The other three social interaction variables evaluated (i.e., parentese speech in a group social context; standard speech in a 1:1 social context; and standard speech in a group social context) were neutral, and unrelated to later word production, controlling for SES.

We replicated this pattern of results in a sample of Spanish-English bilingual families with 11 or 14-month old infants who also wore the LENA recorder for 4 days (Ramírez-Esparza et al., 2017). Overall parentese speech in a 1:1 context (i.e., regardless of the language spoken) was related to concurrent vocalizations and overall productive vocabulary (i.e., number of words produced in Spanish plus number of words produced in English) at 24 months of age, controlling for SES. In addition, language specific correlations were found: parentese speech-1:1 in English was related to productive vocabulary in English at 24 months, but not to productive vocabulary in Spanish; likewise, parentese speech-1:1 in Spanish was related to productive vocabulary in Spanish, but not in English, controlling for SES. In sum, our studies (Ramírez-Esparza et al., 2014, 2017) demonstrate that parentese speech is most beneficial for language learning during infancy when it occurs in 1:1 setting, which we propose promotes contingent bi-directional interactions. Caregivers’ continuous adjustments to their infants’ behavior facilitates exchanges and positive interactions (Saint-Georges et al., 2013).

### Language Learning As a Function of Speech Style and Social Context in Older Children

Although most studies of speech style and social context evaluate prelinguistic children, there are a few studies of older children. Ma et al. (2011) showed differences in novel word learning across speech style in toddlers. Specifically, 21-month-old children were exposed to novel words in parentese speech or standard speech. As a group, the children learned the novel words only when they were presented in parentese speech. However, follow-up analyses showed that 21-month-old children with higher vocabularies were also able to learn the novel words when they were presented in standard-speech. The authors then recruited older children at 27 months of age, and exposed them to the novel words in standard speech only. The authors speculated that because the 21-month-old children with high vocabularies were able to learn the novel words in standard speech, older children might be also able to learn the novel words in standard speech. The results confirmed this expectation.

Differences in novel word learning have also been found in toddlers across social context. Roseberry et al. (2014) recruited 24–30 month old children and exposed them to novel verbs in one of three conditions. In one condition the child learned the novel verbs during a live social interaction with the experimenter, in another condition the child interacted with the researchers via a video chat using Skype, and in the third condition the child was exposed to a yoked video that was prerecorded by the researchers. The results demonstrated that the children were able to learn the novel verbs more efficiently in the contingent interactions (i.e., live social interaction and live video chat) than in the non-contingent social interactions (i.e., yoked video), indicating that older children benefit from contingent bi-directional interactions.

In sum, the studies that have analyzed language learning as a function of speech style and social context in older children demonstrate that 21-month old children who have higher vocabularies can learn from standard speech (Ma et al., 2011), and that while older children are able to learn words from standard speech (Ma et al., 2011) they continue to benefit from contingent bi-directional interactions (Roseberry et al., 2014). Research designed to understand the interactive effects of speech style and social context on language learning in children older than 30 months has not yet been conducted.

### The Present Study

Previous research regarding speech style, social context, and language learning shows that parentese speech and contingent bi-directional interaction are important for language learning in prelinguistic infants, independently (Goldstein et al., 2003; Liu et al., 2003; Goldstein and Schwade, 2008; Singh et al., 2009) and in combination (Braarud and Stormark, 2008; Smith and Trainor, 2008; Ramírez-Esparza et al., 2014, 2017). While the effects of parentese speech on language learning decline with age and advanced vocabulary (Ma et al., 2011), contingent bi-directional interaction continues to impact language learning in toddlers (Roseberry et al., 2014). Less is known about the changes in the characteristics of speech style and social context in language input from infancy to childhood. Although parentese speech is clearly replaced by standard speech as children get older, only a few studies of the acoustic properties of parentese speech in infancy and childhood have been conducted. These studies yielded contradictory results depending on participant age: studies including both infants and children 2 years or older report changes in the use of parentese over time (Stern et al., 1983; Liu et al., 2009); and studies restricted to children either younger than 2 years or older than 2 years report no changes (Warren-Leubecker and Bohannon, 1984; Burnham et al., 2015). The developmental pattern of the social context of language input in natural settings has not yet been studied. In the present study, we evaluate the relationships among speech style, social context and language learning across time. To accomplish this aim we invited families of infant participants who were recruited at 11 or 14 months of age (Ramírez-Esparza et al., 2014, 2017) to participate in this follow-up study when they were 33 months old.^1^

The general goal of the present study was to examine age related changes in the use of parentese speech (identified by acoustic signature and prosodic features) and social context (1:1 and group) as it occurs in natural settings. Specifically, we document age-related changes based on first-person audio recordings of caregivers engaged in natural conversations with their infant, and later with their child, in two social contexts: when the conversation occurs between the child and one adult (1:1) or when the conversation occurs between the child and more than one adult. Then we evaluate the relationships between speech style (parentese speech or standard speech), social context (interactions with one or with more adults) and language learning.

Our study asks three questions: (1) Do the characteristics of language input in terms of speech style and social context change over time? We hypothesize that parentese speech will decrease from infancy to childhood, consistent with the results of previous studies of speech style over time with both infant and toddler participants, and research showing that the effects of parentese speech on language learning decline with age. Although there is no previous research concerning the developmental change in social context across infancy and early childhood, we hypothesize that language input in group context may increase slightly because older children are awake and active for most or all of the recording day, and able to more fully participate in family and community life. However, we hypothesize that language input in 1:1 social context would continue to impact language development based on evidence that contingent bi-directional interaction is important for language learning across the age range of the present study. (2) Do social interaction variables in infancy relate to language development at 33 months of age?

Because our previous studies show that parentese speech in a 1:1 social context relate to language development at 24 months of age, controlling for SES, we expected that parentese speech in a 1:1 context would be related to language development at 33 months of age, controlling for SES. (3) What social interaction variables are related to concurrent language development later in life? Because previous studies of language learning in toddlers demonstrate that standard speech is as effective as parentese speech (Ma et al., 2011) and that contingent bi-directional interactions facilitate learning (Roseberry et al., 2014), we anticipate that standard speech in 1:1 social context will be related to language learning at 33 months of age.

## Materials and Methods

### Participants

We invited the monolingual (Ramírez-Esparza et al., 2014) and Spanish–English bilingual (Ramírez-Esparza et al., 2017) participants who were originally recruited at 11 or 14 months (Time 1) of age as part of a large scale study at the Institute for Learning and Brain Sciences, in Seattle, WA, United States, to participate in this follow-up study (Time 2). Twenty-one of 26 monolingual participants and 14 of 25 bilingual participants who were re-contacted agreed to participate. Language dominance of children originally enrolled in the bilingual study was determined based on the LENA recordings (see *Social Context and Language Activity Assessment* section in Methods below); specifically, word production/use in each language (See *Language Development Assessment* section in Methods below). Of the 14 bilingual participants, 9 were strongly English dominant, producing many more words in English (Mean = 1,448.98, SD = 601.79) than in Spanish (Mean = 25.44, *SD* = 26.85). In addition, the measure of English word production/use in the 9 strongly English dominant bilingual participants was comparable to the 21 monolinguals (Mean = 1,657.98, *SD* = 579.15; *t* = 0.90, *p* = 0.378). The remaining five bilingual participants were more balanced, producing about the same number of words in English (Mean = 385.19, *SD* = 183.45) and in Spanish (Mean = 426.20, *SD* = 207.67). Consequently, analysis was restricted to 21 English monolinguals and the 9 English dominant bilinguals (yielding a sample size of 30 participants) and the measure of English word production/use. This approach increased effect size and avoided the type-1 error typical of small samples (Funder et al., 2014).

Of the sample of 30 children (15 females), 13 had been originally recruited when they were 11 months of age (age range 10 months and 30 days to 11 months and 9 days) and 17 when they were 14 months of age (age range 13 months and 24 days to 14 months and 25 days). Mean age of the children at Time 2 was 33 months of age (age range 31 months and 29 days to 34 months and 9 days). All children were born full-term (37-43 weeks), had normal birth weight (2.5–4.5 kg) and had no major birth or postnatal complications. Socioeconomic status (SES) was assessed using the Hollingshead index (Hollingshead, 2011), a widely used measure producing an overall SES score based on parental education level and occupation (Mean = 53.85, *SD* = 9.86, Range = 16–66). More information about the full sample of monolinguals and bilinguals can be found Ramírez-Esparza et al. (2014, 2017), respectively.

### Social Context and Language Activity Assessment

#### Data Collection

In both the original studies (Time 1) and the current study (Time 2) parents received digital language processors (DLPs) and vests with a chest pocket designed to hold the DLP. At Time 1 parents were instructed to record 8 continuous hours each day for 4 days (2 week days and 2 weekend days), yielding approximately 32 h of recorded audio data from each child. Because children were 33 months old at Time 2, and were awake and active for most or all of the recording day, parents were instructed to record eight continuous hours each day for 2 weekend days, yielding approximately 16 h of recorded audio data for each child. This approach minimized the study demands on returning families and maximized families’ willingness to participate in the follow up study. At Time 1 and Time 2 parents were asked to go about their lives and to complete a daily activity diary, noting the most relevant activities for each day.

#### Data Preparation

The audio data were transferred from the DLP to a computer and analyzed with LENA software, which employs advanced speech-identification algorithms that automatically analyze audio files and produce reports of language activity (see Xu et al., 2009; Oller et al., 2010). The LENA software was used to prepare each participant’s large dataset of recorded audio for further coding of language input, social context, and child’s concurrent word production/use. The audio files were processed using the LENA Advanced Data Extractor Tool (ADEX) in order to efficiently identify intervals with the language activity of interest (i.e., adult speech), and eliminate intervals that did not qualify for analysis. ADEX provides outputs for individual speech segments as short as a fraction of a second and was used to segment each participant’s large dataset of recorded audio into 30-s intervals, as well as to automatically calculate an adult word count for each interval. An 8-h recording yields approximately 600–960 intervals with adult word counts after the data are segmented into 30-s intervals. Intervals with zero adult words are removed and intervals that are at least 3-min apart are selected from the remaining intervals across the entire day, chosen from those with the highest adult word counts. Intervals for coding are identified based on adult word count in order to ensure that there is language activity that will allow coding of social behaviors. Using this approach, we avoid coding when there is no social activity, only silence or noise (e.g., the infant was sleeping, the child wasn’t wearing the recorder). At Time 1, 40 intervals were identified for each participant on each of the 4 days, yielding approximately 160 intervals. Because the children were older at Time 2, and were awake and active for most or all of the recording day, 50 intervals were identified for each participant on each of the 2 days, instead of 40, yielding approximately 100 intervals.

#### Social Environment Coding of Sound Inventory (SECSI)

Ramírez-Esparza et al. (2014, 2017) adapted the Social Environment Coding of Sound Inventory (SECSI, Mehl et al., 2007; Ramírez-Esparza et al., 2009) to assess moment-to-moment naturalistic social behaviors, environments and interactions among caregivers and their children. The SECSI was designed to be a broad system, and allows for coding of wide variety of behaviors, including 73 categories organized into six clusters: “speech partners,” “speech style,” “social context,” “children’s speech utterances,” “activities,” and “mood.” A subset of categories within these clusters was used for the current study to provide social interaction variables. For all data in this study, the following categories were entered into the analysis: “speech partners”—mom speaks to infant, dad speaks to infant, other adult speaks to infant; “speech style”—parentese speech is used to address the infant (i.e., the mother, father and/or other adult uses speech that is higher in pitch, slow in tempo, and has exaggerated intonation contours), standard speech is used to address the infant (i.e., the mother, father or other adult uses ordinary speech); “social context”—infant is with 1 adult, infant is with 2 or more adults.

#### Coding Infant SECSI Categories

Identified intervals (i.e., 160 intervals at Time 1 and 100 intervals at Time 2) were coded for each participant by trained coders. Coders had extensive experience coding parentese, but received additional training in identifying Infant SECSI categories. After training coders were tested independently with a training file to evaluate inter-coder reliability. The reliability analysis produced an average intra-class correlation of 0.91—indicating effective training and reliable coding—based on a two-way random effects model (ICC [2, k]; Shrout and Fleiss, 1979). Furthermore, we independently verified that the intervals coded as parentese vs. standard speech after training contained the acoustic differences characteristic of these two speech styles (i.e., that is, significantly higher pitch and significantly larger pitch range for parentese speech (see Ramírez-Esparza et al., 2014, **Table 1** and Footnote 2 for more information).

**Table 1:** Mean differences between social interaction variables at Time 1 and Time 2.

	Relative Time Use Estimates % Intervals		
	Time 1	Time 2	
Social Interaction Variables (from SECSI)	Mean (*SD*) *n* = 30	Mean (*SD*) *n* = 30	*t*-tests
Parentese Speech-1:1	40.44 (18.42)	5.53 (12.03)	9.74^∗∗∗^
Parentese Speech-Group	18.51 (7.20)	7.39 (15.60)	3.51^∗∗^
Standard Speech-1:1	10.03 (7.49)	33.31 (23.91)	-5.01^∗∗∗^
Standard Speech-Group	18.80 (7.93)	33.41 (17.06)	-5.23^∗∗∗^

*^∗∗^*p* < 0.01 ^∗∗∗^*p* < 0.001.*

*SECSI, Social Environment Coding of Sound Inventory.*

*Time 1 measures were collected in infancy and Time 2 measures were collected in childhood at 33 months of age.*

Coders were provided with basic information about each interval (date, day of the week, time of day, and the time stamp of the audio recording) and the participants’ end-of-day diaries to supplement audio recordings. Transcribing software played the specific 30-s interval for coding based on the time stamp entered. The coders listened to each 30-s interval and coded each SECSI category associated with the interval. In a given 30-s interval the coders entered “YES” if the behavior of interest occurred. The resulting matrix of YES and NO responses indicated that a specific SECSI category occurred or did not occur in that interval. SECSI categories are non-exhaustive and non-mutually exclusive; that is, several SECSI categories could be coded within a single interval (e.g., child is talking, adult talking to physically present others, adult talking to child, adult is using standard speech to address the child, adult is using parentese speech to address child – all within a single 30-s interval). Some intervals were excluded from analyses due to problems noted during coding of the recording (e.g., child was not wearing the recorder, excessive noise). An average of 154.53 (*SD* = 4.82) intervals at Time 1 and an average of 99.8 (*SD* = 0.66) intervals at Time 2 were included in the analyses.

#### Relative Time Use Estimates of SECSI Categories

The coded data matrices containing YES and NO responses for each participant were aggregated to provide relative time use (or proportion of time use) data by calculating the percentage of intervals coded for each category. For example, a relative time use estimate of 45% for the Child SECSI category “Mom speaks to child” indicated that for a participant with 100 intervals, this category was coded YES in 45 of the 100 coded intervals for that participant (see Ramírez-Esparza et al., 2014, 2017).

### Social Interaction Variables Assessment

#### Social Interaction Variables Analyzed in the Study

We examined 4 different social interaction variables based on the SECSI categories used in the analysis at Time 1 and at Time 2. SECSI categories coded at Time 1 refer to interaction occurring between the caregivers and the infant when they were 11 or 14 months of age. SECSI categories coded at Time 2 refer to interactions occurring between caregivers and the child when they were 33 months of age. The social interaction variables are: (1) *Parentese speech-1:1* —mother, father, or other adult spoke directly to the infant/child, parentese speech was used, and only 1 adult voice was recorded during the interval, (2) *Parentese speech-group —*mother and/or father and/or other adult spoke directly to the infant/child, parentese speech was used, and 2 or more adult voices were recorded during the interval, (3) *Standard speech-1:1* —mother, father, or other adult spoke directly to the infant/child, standard speech was used, and only 1 adult voice was recorded during the interval, (4) *Standard speech-group —*mother and/or father and/or other adult spoke directly to the child, standard speech was used, and 2 or more adult voices were recorded during the interval. The coded data were then converted into relative time use estimates by calculating the percentage of valid intervals included in a specific category across all coded intervals (e.g., percentage of intervals coded parentese speech-1:1, percentage of intervals coded standard speech-group). See **Table 1** for means and standard deviations for each of these social interaction variables in infancy (Time 1) and in childhood (Time 2). Also, see the Supplementary Material for additional analyses completed to compare means of the weekend and weekday intervals at Time 1 with the weekend intervals at Time 2.

### Language Development Assessment

Our previous studies used the MacArthur-Bates Communicative Development Inventory (or CDI; Fenson et al., 2007) Words and Sentences long form to assess language development. However, this instrument is designed for use with 16- to 30-month old children. In the current study of 33-month old children, we used an alternative approach. Specifically, for each of the 100-Time 2 intervals, coders transcribed the child’s speech. Counting the number of intelligible English words produced by the child, and summing across all intervals, yielded a measure of word production/use in English with substantial variability. The children produced an average of 1,595.29 words (*SD* = 583.71). Word production/use was converted to *Z*-scores for use in analysis, and all scores fell within +2.5 standard deviations of the mean.

## Results

### Initial Analyses

The initial step in analysis was evaluation of the overall effects of age group at enrollment (i.e., 11 months vs. 14 months old) on the other experimental variables (SES, word production/use at 33 months, and social interaction variables at Time 1 and Time 2). Participants enrolled in the study at 11 or 14 months showed no significant effects due to age at enrollment for social interaction variables at Time 1 and Time 2, word production/use at 33 months, or SES (see Supplementary Table S3 for means and standard deviations). Participants were collapsed across age at enrollment for the remaining analyses.

In our previous studies of the full sample of monolinguals and bilinguals we found that parentese speech in a 1:1 social context during infancy correlates significantly with socioeconomic status (SES). Therefore, we expected that we would replicate findings for parentese speech-1:1 at Time 1 for the subset enrolled in the current study. Indeed, when we examined relationships between SES (Hollingshead, 2011) and measures of language input (i.e., social interaction variables derived from SECSI), SES was found to be significantly correlated the percent intervals coded for parentese speech in a 1:1 social context at Time 1 (*r* = 0.46, *p* < 0.01, *N* = 30). No other significant correlations were found with SES at Time 1 or Time 2. We also examined the relationship between SES and word production/use at 33 months, and the correlation was not significant (*r* = 0.15, *N* = 30). As in our previous studies (Ramírez-Esparza et al., 2014, 2017) all other correlational analyses were controlled for SES.

### Do the Characteristics of Language Input in Terms of Speech Style and Social Context Change Over Time?

In order to investigate changes in the pattern of the relative time use estimates of social interaction variables from infancy to childhood, we first performed paired *t*-tests to evaluate overall mean differences between the specific variables (**Table 1**). We also performed partial correlations, controlling for SES between social interaction variables in infancy and childhood (**Table 2**).

**Table 2:** Correlations among social interaction variables assessed in infancy (Time 1) and social interaction variables assesses in childhood (Time 2), controlling for SES.

	*Social Interaction Variables assessed in Childhood at Time 2 (from SECSI)*			
*Social Interaction Variables assessed in Infancy at Time 1 (from SECSI)*	Parentese Speech-1:1 *N* = 30	Parentese Speech-Group *N* = 30	Standard Speech-1:1 *N* = 30	Standard Speech-Group *N* = 30
Parentese Speech-1:1	0.15	0.08	0.39^∗^	-0.23
Parentese Speech-Group	-0.06	-0.02	-0.16	0.29
Standard Speech-1:1	-0.15	-0.16	-0.06	0.14
Standard Speech-Group	-0.23	-0.17	-0.25	0.44^∗^

*^∗^*p* < 0.05.*

*SECSI, Social Environment Coding of Sound Inventory.*

**FIGURE 1:**
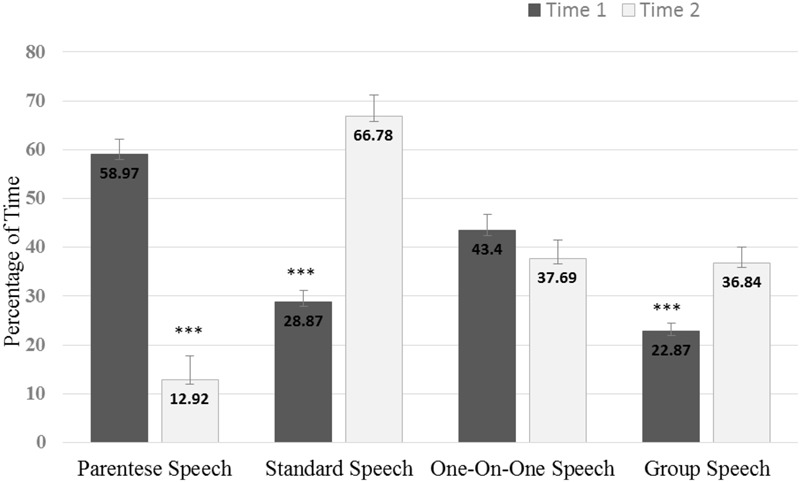
Components of social interaction at Time 1 (when children were 11 or 14 months of age) and at Time 2 (when children were 33 months of age). ^∗∗∗^*p* < 0.001.

Paired t-tests demonstrated that the relative time use estimates of all social interaction variables change significantly across time (see **Table 1** and also see the Supplementary Material for additional analyses completed to compare means of the weekend and weekday intervals at Time 1 with the weekend intervals at Time 2). Both parentese speech-1:1 and parentese speech-group decrease significantly from infancy to childhood. On the other hand, standard speech-1:1 and standard speech-group increase significantly from infancy to childhood. The independent contributions of speech style (i.e., parentese speech and standard speech) and social context (i.e., 1:1 and group) to this pattern of results was also evaluated over time. As seen in **Figure 1**, parentese speech decreased significantly from infancy to childhood (*t* = 8.67, *p* < 0.001), while standard speech (*t* = –7.98, *p* < 0.001) and group social context (*t* = –4.75, *p* < 0.001) increased significantly from infancy to childhood. One-on-one social context is unchanged (*t* = 1.72, *p* = 0.10) across time from infancy to childhood.

Association of social interaction variables assessed at Time 1 and Time 2, controlling for SES, reveals significant positive correlations between parentese speech-1:1 in infancy and standard speech-1:1 in childhood (*r =* 0.39, *p* < 0.05, *df =* 27), and between standard speech-group in infancy and in childhood (*r =* 0.44, *p* < 0.05, *df = 27*). Other variables were not significantly correlated.

### Do Social Interaction Variables in Infancy Relate to Language Development at 33 months of Age?

**FIGURE 2:**
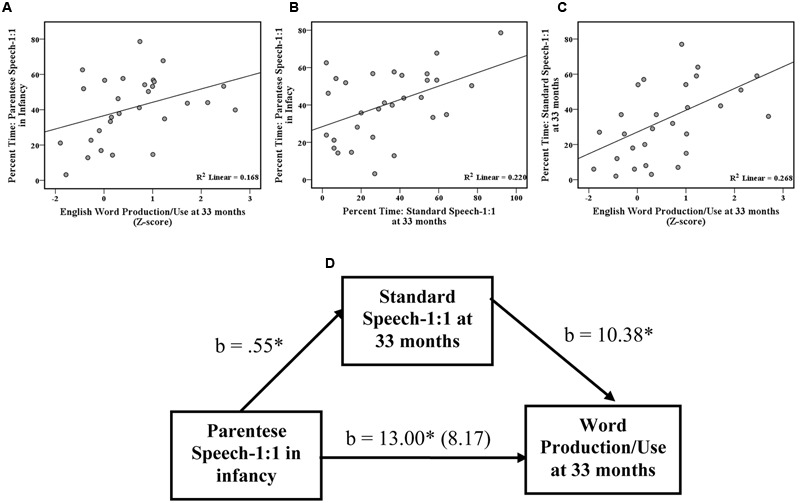
Scatter plots showing the relationships between **(A)** parentese speech-1:1 in infancy and English word production/use at 33 months, **(B)** parentese speech-1:1 in infancy and standard speech-1:1 at 33 months, **(C)** standard speech-1:1 at 33 months and word production/use at 33 months, and **(D)** the mediation analyses showing that standard speech-1:1 in childhood mediates the relationship between parentese speech-1:1 in infancy and word production/use at 33 months. Sample size = 30 infants; b = indicates the regression coefficient; ^∗^*p* < 0.05.

In order to investigate relationships between social interaction variables assessed in infancy and language development at 33 months, we evaluated the associations between word production/use at Time 2 and the 4 social interaction variables measured at Time 1: (1) parentese speech-1:1, (2) parentese speech-group, (3) standard speech-1:1 and (4) standard speech-group using partial correlations controlling for SES. As shown in **Table 3**, Time 1 parentese speech-1:1 was associated with word production/use at 33 months of age (*r =* 0.39, *p* < 0.05, *df =* 27), controlling for SES (also see **Figure 2** for scatterplots of the raw data). No other significant associations were found.

**Table 3:** Correlations between social interaction variables and language development, controlling for SES.

	Language Development
	Word production/use at 33 months
*Social Interaction Variables (from SECSI) at Time 1*	*N* = 30
Parentese Speech-1:1	0.39^∗^
Parentese Speech-Group	-0.01
Standard Speech-1:1	0.14
Standard Speech-Group	-0.12

*Social Interaction Variables (from SECSI) at Time 2*	*N* = 30

Parentese Speech-1:1	-0.20
Parentese Speech-Group	-0.36^+^
Standard Speech-1:1	0.50aaa
Standard Speech-Group	-0.15

*^+^*p* < 0.06, ^∗^*p* < 0.05, aaa*p* < 0.01.*

*Social interaction variables at Time 1 were assessed when children were 11 or 14 months of age and social interaction variables at Time 2 were assessed when children were 33 months of age.*

*SECSI, Social Environment Coding of Sound Inventory.*

### What Social Interaction Variables are Related to Concurrent Language Development Later in Life?

To investigate which social interaction variables are related to concurrent language development later in life, we evaluated the associations between word production/use and the 4 social interaction variables measured at Time 2: (1) parentese speech-1:1, (2) parentese speech-group, (3) standard speech-1:1 and (4) standard speech-group using partial correlations controlling for SES. Standard speech-1:1 was associated with word production/use at 33 months of age (*r =* 0.50, *p* < 0.01, *df =* 27), controlling for SES (**Table 3**, see **Figure 2** for scatter plots of raw data). Furthermore, parentese speech-group was marginally negatively associated with word production/use at 33 months of age (*r =* –0.36, *p* < 0.06, *df =* 27), controlling for SES. No other significant associations were found.

Because parentese speech-1:1 in infancy is itself positively correlated with standard speech-1:1 in childhood, controlling for SES, *r =* 0.39, *p* < 0.05, *df =* 27 (**Table 3**, see **Figure 2** for scatter plots of raw data), we used statistical mediation analysis to examine whether the relationship between parentese speech-1:1 in infancy and word production/use at 33 months of age is mediated by standard speech-1:1 in childhood. Following the guidelines of Baron and Kenny (1986), we found: (1) a significant relationship (*r =* 0.39, *p <* 0.05, *df =* 27) between the predictor variable (parentese speech-1:1 in infancy) and the outcome variable (word production/use at 33 months of age), (2) a significant relationship (*r =* 0.39, *p <* 0.05, *df =* 27) between the predictor variable (parentese speech-1:1 in infancy) and the potential mediator (standard speech-1:1 in childhood), and (3) a significant relationship (*r =* 0.50, *p* < 0.01, *df =* 27) between the mediator variable (standard speech-1:1 in childhood) and the outcome variable (word production/use at 33 months of age), controlling for SES (see **Figure 2**, for scatterplots of raw data). Using the moderated mediation macro by Hayes’ Process (Hayes, 2013), with 1000 bootstrapping re-samples and introducing SES into the model as a covariate, the results show that the relationship between parentese speech-1:1 in infancy and word production/use at 33 months of age is reduced in magnitude when standard speech-1:1 in childhood is included in the model (i.e., from 13.00, *p* < 0.05 to 8.17, *p* =0.21). Standard speech-1:1 in childhood was deemed a significant mediator because the 95% bias-corrected confidence interval did not include zero (i.e., 9363 to 14.16). The partial effect of the control variable SES on word production/use at 33 months of age was not significant (–5.7, *p* =0.61). The final model explained 31% of the variance of word production/use at 33 months (**Figure 2**).

## Discussion

This study asked three questions: (1) Do the characteristics of language input by caregivers to their children in terms of speech style and social context change over time? (2) Does parentese speech in a 1:1 social context in infancy relate to words produced at 33 months of age? (3) What types of social interaction variables are related to concurrent language development later in life? Briefly, our results demonstrate that the characteristics of language input do change over time, with significant differences in all four social interaction variables based on speech style and social context. These changes reflect significant decreases in parentese speech and significant increases in group social context. In addition, the specific social interaction variable associated with language development at 33 months varies over time; that is, only parentese speech-1:1 in infancy was found to be related to language development at 33 months, and only standard speech-1:1 in childhood was found to be related to concurrent language development.

### Do the Characteristics of Language Input in Terms of Speech Style and Social Context Change Over Time?

Results showed that the use of parentese speech-1:1 and parentese speech-group by caregivers diminishes significantly from infancy to childhood. In contrast, caregivers significantly increase the use of standard speech-1:1 and standard speech-group. These differences were driven by a significant reduction in parentese speech and a significant increase in standard speech. Specifically, parentese speech in childhood is reduced by 78% compared to parentese speech in infancy (i.e., from 59% of coded intervals in infancy to 13% of coded intervals in childhood). And, standard speech more than doubles from infancy to childhood (i.e., from 29% in infancy to 67% in childhood). Future investigations using acoustic analyses of natural conversations would provide a more comprehensive picture of parentese speech in natural settings, and could indicate whether the previously reported pattern of decreasing acoustic exaggeration in speech directed to infants, children and adults is also observed in recordings from daily life (Stern et al., 1983; Liu et al., 2009).

Our finding of a small but significant increase in group interactions from infancy to childhood (i.e., from 23 to 37%), is consistent with our speculation that as children grow older, they are more able to participate in day-to-day family life resulting in more frequent group interaction. It is also possible that this change is driven by the presence of other children in the home. Interestingly, although 1:1 speech decreased slightly from infancy to childhood (i.e., from 43 to 38%), this decrease does not reach significance. This finding may indicate that the pattern of 1:1 interactions that caregivers establish with their children during infancy is maintained in childhood (as discussed below). In fact, the correlations between social context variables assessed in infancy and in childhood, controlling for SES, support this argument: Caregivers who use more parentese speech in a one-on-one context in infancy are likely to use more standard-speech in a one-on-one context in childhood. In other words, caregivers change speech style across time, but maintain social context—continuing patterns of contingent bi-directional interactions across time.

### Do Social Interaction Variables in Infancy Relate to Language Development at 33 months of Age?

We expected parentese speech-1:1 to be related to total word production/use at 33 months, and other social interaction variables to be unrelated to language development, controlling for SES. Our findings confirmed this expectation, and were consistent with our previous studies demonstrating that parentese speech-1:1 is related to productive vocabulary at 24 months in monolinguals (Ramírez-Esparza et al., 2014) and in bilinguals (Ramírez-Esparza et al., 2017). As in our earlier work, the effects of parentese speech 1:1 on later word production/use were substantial: children with the highest amount of parentese speech-1:1 in infancy (*>2 SD, N = 7*) produced an average 400 more words at 33 months than children with the lowest amount of parentese speech-1:1 in infancy (*<*2 *SD, N = 8*), mean word count = 1,653.34 vs. 1,206.42, respectively. This finding further supports the idea that parentese speech in a 1:1 social context facilitates contingent bi-directional interactions between caregivers and their infants.

The importance of parentese speech in infancy may be explained by the fact that this type of speech helps infants to construct phonetic categories (Kuhl et al., 1997). Parentese speech also provides clarity for consonants. For example, Cristia (2011) found that 4- to 6- month old infants and 12- to 14 old infants whose caregivers produced a more acoustically extreme /s/ were able to discriminate this category from another sound. This kind of clear, hyper-articulated speech is beneficial for infants as they learn the sounds of their native language, but less important as they get older. Also, when caregivers use parentese speech in 1:1 interactions, infants are able to direct their attention to those sounds and, in turn, the caregivers are sensitive to the positive response from their infants (Braarud and Stormark, 2008; Smith and Trainor, 2008). This contingent bi-directional interaction therefore supports language learning (e.g., Goldstein et al., 2003; Goldstein and Schwade, 2008; Ramírez-Esparza et al., 2014, 2017).

### What Social Interaction Variables are Related to Concurrent Language Development Later in Life?

Based on previous work we inferred that standard speech in a 1:1 setting would be related to word production/use at 33 months of age, controlling for SES. Our findings confirmed this expectation. Furthermore, no other concurrent social interaction variables were significantly related to word production/use at 33 months of age, including parentese speech-1:1. The fact that standard speech-1:1 was the only variable related to concurrent language development is consistent with our finding that caregivers in this study decreased the use of parentese speech and increased the use of standard speech over time. It supports previous work showing that standard speech is as effective as parentese speech in lab based tasks assessing novel word learning in older children (Ma et al., 2011). It is also consistent with previous work showing that contingent bi-directional interactions have a positive effect on language learning in toddlers (e.g., Roseberry et al., 2014).

The mediation analyses showed that standard speech-1:1 in childhood fully mediated the effects of parentese speech-1:1 in infancy on language development, controlling for SES. These analyses demonstrate that children who experience increased parentese speech in a 1:1 setting in infancy also experience increased standard speech in a 1:1 setting in childhood. Caregivers, and particularly other adult caregivers, may change over time but it appears that all caregivers naturally migrate from parentese to standard speech as children grow older; however, continued engagement with their children in a 1:1 social context is positively related to concurrent word production/use, an effect that may be due to contingent bi-directional interaction. Because our studies are based in the United States, future investigations would benefit from replicating these findings in other cultures. For example, Western middle class mothers tend to consider their children to be conversational partners (Lieven, 1994; Hoff, 2006); however, in other cultures, such as the Mayan in Mexico, children are considered passive observers of the language around them, not active conversational partners (Shneidman and Goldin-Meadow, 2012). Investigating the effects of contingent bi-directional interactions in other cultures and with children learning two languages (e.g., Ramírez-Esparza et al., 2017) would be of great interest.

### Limitations and Future Directions

In this investigation, we argue that contingent bi-directional interactions are favorable for language learning. However, these findings are preliminary and only explain 31% of the variance. There are other possible explanations. For example, joint attention, or the infants’ ability to coordinate their attention between a person and an object has been found to be related to language learning (e.g., Benigno et al., 2007; Conboy et al., 2015). Other studies have also shown that the words used by the parents are related to language learning. For example, Shneidman et al. (2013) find that the number of word tokens directed to the child by a primary caregiver and other members of the household at 2.5 years of age is related to the Peabody Picture Vocabulary Test (PPVT), a measure of receptive vocabulary. Similarly, Rowe (2012) reported that the total number of word tokens, the total number of different word types (or vocabulary diversity), and the total number of different rare words (or vocabulary sophistication) was related to the PPVT. Thus, for future studies it is important to consider not only the characteristics of the social context, but also the characteristics of the speech used by caregivers.

The method used to assess children’s word production/use (i.e., counting the words produced by the child in the selected intervals) may be impacted by talkativeness. Furthermore, it is also possible that caregivers engage in 1:1 interactions and/or use parentese speech with children who are more “talkative” or social, and are less likely to engage in these behaviors with children who less “talkative” or less social (Locke, 2006). For example, Fischer and colleagues (Fischer et al., 2011) found that caregivers use parentese-speech when talking to their infants, but use a more standard-like speech when they talk to a “babyface” robot. We did not select intervals on the basis children’s talkativeness, and therefore cannot evaluate the role of children’s talkativeness in measures of word production/use or social interaction variables. These questions are of interest for future work.

Another limitation of the present study is the inclusion of strongly English dominant children from families who identified themselves as bilingual (i.e., *N* = 9) when their children were infants. Although their word production/use at 33 months did not differ significantly from the monolinguals, there may be other differences in conversation patterns due to cultural values (Ramírez-Esparza et al., 2017). Future studies are needed in larger and more diverse samples of bilinguals in order to test the variables used in this study as a function of bilingual language experience.

## Conclusion

The general goal of this study was to investigate changes in caregiver–child social interactions in terms of speech style and social context, and evaluate relationships between social interaction variables and language learning across time, from infancy to childhood. We used the LENA system to record everyday conversations and word production/use in children in natural settings at 33 months and compared results to identical measures collected in previous studies (Ramírez-Esparza et al., 2014, 2017). We found that the dominant speech style caregivers used changed as the child developed, from usage of parentese speech in infancy to standard speech in childhood, and that this developmental change had consequences for the relations between social interaction variables and language learning. Our results showed that parentese speech in a 1:1 setting in infancy is related to later language learning at 24 and 33 months, and that standard speech-1:1 at 33 months is related to concurrent word production/use. Furthermore, parentese speech-1:1 in infancy is correlated with standard speech-1:1 in childhood, and standard speech-1:1 in childhood fully mediated the effects of parentese speech-1:1 in infancy on language development. This suggests that the effects of 1:1 interactions persist even when a natural developmental timeline replaces parentese speech with standard speech. The persistence of the importance of 1:1 social interactions over time indicates the importance of contingent bi-directional interactions on language development. The findings suggest that if parents engage in quality 1:1 social interactions with their children they will be likely be pleased about who’s talking to them NOW!

## Ethics Statement

The study was approved by the ethics committee of University of Washington.

## Author Contributions

NR-E collected the data, analyzed the data, and wrote the manuscript. AG-S collected the data, and co-wrote the manuscript. PK supervised the project, and co-wrote the manuscript.

## Conflict of Interest Statement

The authors declare that the research was conducted in the absence of any commercial or financial relationships that could be construed as a potential conflict of interest.

## Footnotes

1 We planned to follow-up infant participants approximately 2 years after enrollment in the Time 1 studies. In this large-scale longitudinal study, families were invited to complete additional measures every 3 months until children were 33 months old. Measures collected between enrollment and 30 months of age were beyond the scope of this paper. The results of the final follow-up assessment at 33 months are reported here.
