# Supplementary material for: Look Who’s Talking NOW! Parentese Speech, Social Context, and Language Development Across Time
Source: Front Psychol. 2017 Jun 20;8:1008. doi: 10.3389/fpsyg.2017.01008 (PMC5477750; doi:10.3389/fpsyg.2017.01008)
Supplement: Supplementary file 1 [file Table_1.pdf]

Supplementary Material

Look Who's Talking NOW! Parentese Speech, Social Context and Language Development Across Time

Nairán Ramírez-Esparza*, Adrián García-Sierra, Patricia K. Kuhl

*** Correspondence:** Nairán Ramírez-Esparza: nairan.ramirez@uconn.edu

1 Supplementary Data

Additional analyses were completed to compare means of the weekend intervals and weekday intervals at Time 1 (in infancy) with the weekend intervals at Time 2 (in childhood). These results show results consistent with the full data set (see Table 1 in the manuscript). Means are significantly different for all social interaction variables in infancy (Time 1) and in childhood (Time 2) when the comparison is restricted to weekend intervals at Time 1 (Supplementary Table 1). In addition, means are significantly different for all social interactions variables in infancy (Time 1) and in childhood (Time 2) when the comparison is restricted to weekend intervals at Time 1, except for one variable: parentese-speech group (Supplementary Table 2). This exception is due to the fact that at Time 1, there is less parentese-speech group during weekdays (13.33%) than weekends (23.21%).

Supplementary Table 1: Mean values for social interaction variables in infancy at Time 1 (weekend recordings only) and in childhood at Time 2 (weekend recordings)

	Relative Time Use Estimates % Intervals		
	Time 1-weekend	Time 2-weekend	
Social Interaction Variables	Mean (SD) n = 30	Mean (SD) n = 30	t-tests
Parentese Speech-1:1	31.52 (19.19)	5.53 (12.03)	7.64***
Parentese Speech-Group	23.24 (7.11)	7.39 (15.60)	4.42***
Standard Speech-1:1	8.11 (7.49)	33.31 (23.91)	-5.66***
Standard Speech-Group	24.95 (12.51)	33.41 (17.06)	-2.60*

* p < .05 *** p < .001

Note: Time 1 measures were collected in infancy and Time 2 measures were collected in childhood at 33 months of age.

Supplementary Table 2: Mean values for social interaction variables in infancy at Time 1 (weekdays only) and in childhood at Time 2 (weekend recordings)

	Relative Time Use Estimates % Intervals		
	Time 1-weekdays	Time 2-weekends	
Social Interaction Variables	Mean (SD) n = 30	Mean (SD) n = 30	t-tests
Parentese Speech-1:1	48.41 (24.33)	5.53 (12.03)	8.79***
Parentese Speech-Group	13.33 (9.28)	7.39 (15.60)	1.79
Standard Speech-1:1	11.70 (10.11)	33.31 (23.91)	-4.35***
Standard Speech-Group	12.35 (9.28)	33.41 (17.06)	-7.11***

* p < .05 *** p < .001

Note: Time 1 measures were collected in infancy and Time 2 measures were collected in childhood at 33 months of age.

2 Supplementary Table

Supplementary Table 3: Mean values for SES, word production/use and social interaction variables at in infancy at Time 1 and in childhood at Time 2 as a function of age at enrollment.

	Age at enrollment		
	11-months	14-months	
Variables	Mean (SD) n = 13	Mean (SD) n = 17	t-tests
SES	55.15 (5.78)	52.85 (12.19)	.40
Parentese Speech-1:1 (Time 1)	42.94 (20.05)	38.53 (17.45)	.64
Parentese Speech-Group (Time 1)	19.73(7.94)	17.57(6.69)	.43
Standard Speech-1:1 (Time 1)	10.65(9.89)	9.55(5.26)	.39
Standard Speech-Group (Time 1)	19.87(8.83)	17.98(7.33)	.64
Parentese Speech-1:1 (Time 2)	1.24 (1.90)	8.81 (15.27)	-1.77
Parentese Speech-Group (Time 2)	1.7 (4.66)	11.75 (19.45)	-1.82
Standard Speech-1:1 (Time 2)	40.78 (23.78)	27.59 (23.07)	1.53
Standard Speech-Group (Time 2)	35.19 (19.23)	32.05 (15.66)	.49
Word production/use (Time 2)	1645.20 (520.50)	1557.12(640.92)	.63

Note 1: none of the t-tests are significant at the .05 level.

Note 2: Time 1 measures were collected in infancy and Time 2 measures were collected in childhood at 33 months of age.
